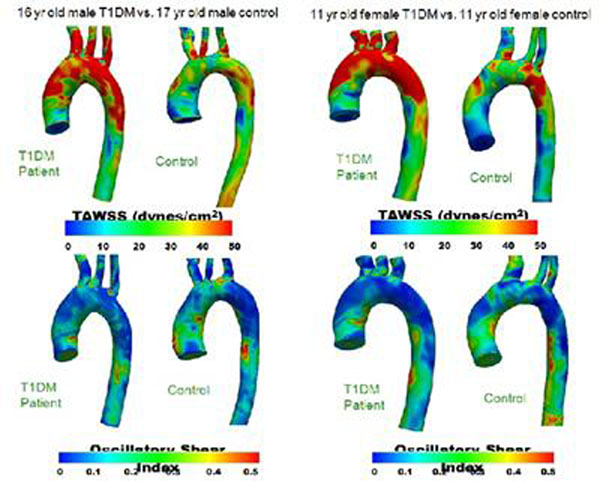# Cardiac magnetic resonance imaging can detect early vascular changes in children with type 1 diabetes (T1DM)

**DOI:** 10.1186/1532-429X-13-S1-P381

**Published:** 2011-02-02

**Authors:** Margaret M Samyn, Pippa Simpson, Michael E Widlansky, Mary Krolikowski, Jennifer G Co-Vu, Ronak Dholakia, John F LaDisa, Ramin Alemzadeh

**Affiliations:** 1 Medical College of Wisconsin, Milwaukee, WI, USA; 2 Children's Hospital of Wisconsin, Milwaukee, WI, USA; 3 Marquette University, Biomedical Engineering, Milwaukee, WI, USA

## 

Adult studies have shown that cardiac magnetic resonance (CMR) can image vascular changes including altered aortic compliance and early plaque which correlates well with Framingham risk score and endothelial function. Impaired endothelial function, measured with brachial artery reactivity testing, is recognized as an early and modulating process in the pathophysiology of atherosclerotic plaque development. No CMR imaging studies of early atherosclerosis and vascular health exist in diabetic pediatric populations. This prospective pilot CMR proof of concept study hypothesized that children with T1DM will have thoracic aortic wall characteristics, as well as CMR-derived aortic computational fluid dynamic (CFD) models, different from age-matched control subjects. Furthermore, a positive correlation was sought between key CMR data and brachial artery reactivity measures, as well as cardiac venous biomarkers in T1DM. 7 control and 8 T1DM pediatric subjects had same-day fasting CMR scan, brachial artery testing, and venous blood draw (for lipid panel, HgbA1c, glucose, high sensitivity c-reactive protein (hs-cRP), fibrinogen, and homocysteine). Enrolled T1DM and control subjects were similar: age (14.5 ±1.7 versus (vs.) 15.2 ± 2.7 years), sex (4 male and 4 female T1DM vs. 5 male and 2 female controls), weight (55.7 ± 14.8 vs. 67.6 ± 23.5 kg), body mass index (20.8 ± 4.3 vs. 23.1 ± 5.7 kg/m^2^) and systolic blood pressure (113 ± 11.4 vs. 116 ± 8.8). They had no significant differences in lipid values, fibrinogen, or hs-cRP, but did differ with regard to glucose (182.1 ± 100.8 vs. 85.4 ± 5.7, p < 0.02), HgbA1c (9.0 ± 2.2 vs. 5.2 ± 0.3, p < 0.001), and homocysteine (4.3 ± 0.6 vs. 5.7 ± 1.0, p < 0.04). While brachial artery reactivity did not change with age, CMR determined ascending aortic (AAo) compliance decreased with increasing age in T1DM (R^2^ = 0.54, p < 0.09), but not for controls. In T1DM, CMR determined ascending aortic (AAo) compliance declined, as hs-cRP increased (R^2^ = 0.66, p < 0.05).No other significant correlations existed between AAo compliance and venous biomarkers. Preliminary qualitative analyses of aortic CFD models show different patterns of aortic wall shear stress and oscillatory shear index (OSI) for T1DM versus age-matched control pediatric subjects (Figure 1). In conclusion, this pilot CMR study of T1DM and control pediatric subjects illustrates that early differences in vascular characteristics can be detected by MRI and correlate with age and with select venous biomarkers.

**Figure 1:**